# Trends in treatment of retinal disorders in the Brazilian Public Health System over a 10-year period^*^

**DOI:** 10.31744/einstein_journal/2021GS6616

**Published:** 2021-11-24

**Authors:** Aline Nunes Ferraz, Rafael da Silva Lemos, Fernando Korn Malerbi, Rodrigo Brant, Arthur Gustavo Fernandes

**Affiliations:** 1 Universidade Federal de São Paulo Escola Paulista de Medicina São Paulo SP Brazil Escola Paulista de Medicina, Universidade Federal de São Paulo, São Paulo, SP, Brazil.

**Keywords:** Retinal diseases/epidemiology, Intravitreal injections, Light coagulation, Public health surveillance, Epidemiology, Health care costs

## Abstract

**Objective:**

To investigate trends in terms of number and cost of intravitreal injection, photocoagulation and panphotocoagulation procedures performed by the Brazilian Public Health System, from 2010 to 2019.

**Methods:**

The Brazilian Public Health System Database was used as the primary source of data. Intravitreal injection, photocoagulation and panphotocoagulation procedures performed from 2010 to 2019 were investigated. Procedure prevalence and cost trends were analyzed according to year and region. Annual trends were examined using generalized linear models, with a significance level of 5% (p=0.05).

**Results:**

There was a significant increase in the prevalence of intravitreal injections (1,088%), panphotocoagulation (51%) and photocoagulation (37%) procedures from 2010 to 2019. Intravitreal injections accounted for the most significant increase. However, costs were not significantly readjusted over the years.

**Conclusion:**

Over a 10-year period, there was a significant increase in the number of procedures associated with retinal disorders. Procedure costs saw little readjustments over time. In spite of limitations, inaccuracies and lack of details, the Brazilian Public Health System Database is the primary source of data for the Public Health System related research in Brazil, and can contribute with information on ocular health and costs of ophthalmic procedures.

## INTRODUCTION

Intravitreal injections, indicated for age-related macular degeneration (AMD) and macular edema; photocoagulation, indicated for retinal tears; and retinal panphotocoagulation, indicated in cases of proliferative diabetic retinopathy, macular edema and retinal vein occlusion, among other diseases, are the most common procedures used by vitreoretinal specialists to treat retinal disorders.^(1,2)^

Therapeutic retinal photocoagulation has been used for more than 50 years^(3)^ and is prescribed for all cases of microaneurysms and other focal leakage sites in the macula area.^(4)^ A recent meta-analysis revealed that photocoagulation as a single therapy can reduce the chances of visual loss within one to three years compared to no intervention.^(3)^ Panphotocoagulation is defined as total retinal laser ablation combined with small scattered focal ablations, spots aimed to prevent further vessel growth and leakage, due to secretion of vascular endothelial growth factor (VEGF) by the ischemic retina.^(2)^

Lasers have been the primary treatment modality for patients with macular edema for several years. However, the introduction of intravitreal injections has revolutionized the therapeutic management of this disorder in the past decades. Recent reviews have shown the success of therapeutic intravitreal injections for visual function improvement in patients with macular edema. In these studies, higher injection frequency and younger age were the main variables for better visual prognosis and superior treatment outcomes.^(5)^

Brazil is one of the few countries worldwide to offer universal, free health coverage financed by the federal government Public Health System (*Sistema Único de Saúde* - SUS). This system provides medical attention to the Brazilian population in every medical specialty, from primary healthcare to complex procedures delivered by tertiary hospitals, including eye care. National data derived from SUS are stored in the Public Health System Database (*Departamento de Informática do Sistema Único de Saúde* - DATASUS).^(6)^ Originally intended for administrative procedures, this database contains data on all hospital admissions and procedures covered by SUS in Brazil. The online version protects personal information, but provides access to data associated with specific procedure codes, general demographic information, place and date of admission and procedure costs.^(7)^

## OBJECTIVE

To investigate trends in terms of number and cost of intravitreal injection, photocoagulation, and panphotocoagulation procedures, performed by the Brazilian Public Health System, from 2010 to 2019.

## METHODS

The DATASUS was the primary source of data in this study. This database is an initiative of the Brazilian Federal Government aimed to collect data from the national health system and contains information gathered from all public hospitals in the country.

Intravitreal injection, photocoagulation and panphotocoagulation procedures performed by the SUS, between 2010 and 2019, were included in this study. Prevalence of procedures and costs were analyzed per year and region.

Prevalence was calculated as the total number of procedures performed in the country in a given year, according to the respective population census published by the Brazilian Institute of Geography and Statistics (*Instituto Brasileiro de Geografia e Estatística* - IBGE).

Statistical analyses were performed using Stata/SE Statistical Software, version 14.0, 2015 (Stata Corp, College Station, Texas, United States). Frequency tables were used for descriptive analysis. Annual trends were examined using generalized linear models. P values ≤0.05 were considered statistically significant.

## RESULTS

**Figure 1:**
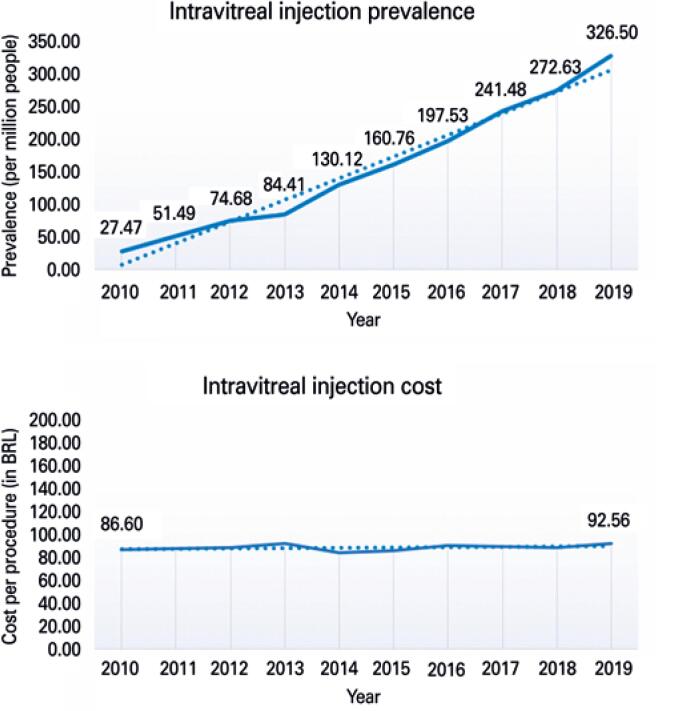
Intravitreal injection prevalence and cost trends from 2010 to 2019

Over the course of the 10-year period (2010 to 2019), 322,046 intravitreal injections were performed by the SUS. Figure 1 shows prevalence trends per 1 million people, and costs per procedure during these years.

Trend analysis showed a significant (p<0.001) increase in prevalence over time. Comparisons between 2019 and 2010 revealed a 1,088.0% difference. Procedure costs practiced by health service providers did not change significantly during the 10-year period (p=0.233).

Table 1 shows the prevalence of intravitreal injections according to region.

**Table 1:** Prevalence (per million people) of intravitreal injections according to region

Year	Region					
	North	Northeast	Southeast	South	Midwest	All
2010	4.99	22.56	29.30	41.94	32.89	27.47
2011	8.37	44.18	64.90	50.56	53.27	51.49
2012	9.20	66.56	97.84	56.68	82.54	74.68
2013	14.55	73.91	106.18	93.97	61.55	84.41
2014	16.44	124.02	160.43	124.22	122.89	130.12
2015	30.47	128.36	215.79	129.91	177.88	160.76
2016	40.87	131.18	282.36	173.28	189.93	197.53
2017	39.43	174.02	346.38	206.73	199.11	241.48
2018	46.91	184.60	382.19	275.53	235.55	272.63
2019	72.92	219.32	463.11	356.05	193.47	326.50

An overall increase in prevalence was observed in all regions of the country, albeit at different rates. Comparisons between 2010 and 2019 revealed an increase in prevalence by 1,481%, 1,363%, 872%, 749% and 488%, in the Southeast, North, Northeast, South and Midwest, respectively.

**Figure 2:**
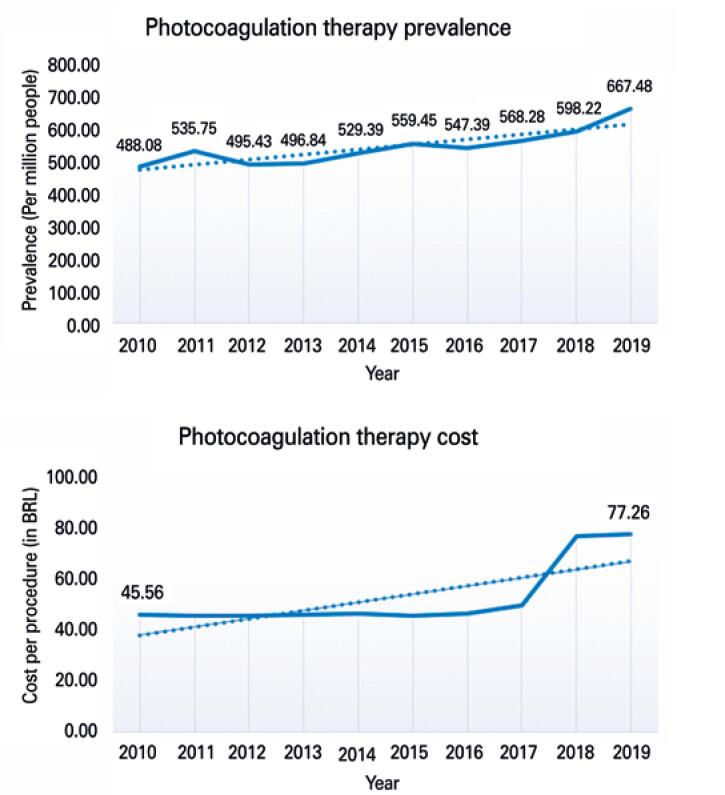
Photocoagulation procedure prevalence and cost trends from 2010 to 2019

Over the course of the 10-year period (2010 to 2019), 1,113,516 photocoagulation procedures were performed by the SUS. Figure 2 shows prevalence and cost trends per 1 million people during this period.

Trend analysis showed a significant increase in prevalence over time (p<0.001). Comparisons between 2019 and 2010 revealed a 37% difference. Significant changes (p=0.015) in costs of procedure performed by health service providers were observed only after 2018.

Table 2 shows the prevalence of photocoagulation procedures according to region.

**Table 2:** Prevalence (per million people) of photocoagulation procedures according to region

Year	Region					
	North	Northeast	Southeast	South	Midwest	All
2010	235.99	397.41	669.70	386.05	274.80	488.08
2011	177.32	373.68	756.73	474.47	409.97	535.75
2012	166.56	323.24	738.53	434.55	249.64	495.43
2013	133.81	281.44	768.05	471.83	224.46	496.84
2014	137.85	222.88	836.75	633.48	177.04	529.39
2015	177.85	193.27	918.93	618.74	208.64	559.45
2016	284.04	211.33	889.40	536.65	183.41	547.39
2017	346.46	222.46	891.41	618.83	182.72	568.28
2018	643.43	237.58	789.48	888.25	240.27	598.22
2019	715.53	257.59	907.37	931.51	262.13	667.48

An overall increase in prevalence was observed in some regions of the country, albeit at different rates. Comparisons between 2010 and 2019 revealed an increase in prevalence in the North, South and Southeast regions (203%, 141% and 35%, respectively) and a decrease in the Northeast and Midwest (35% and 5%, respectively).

**Figure 3:**
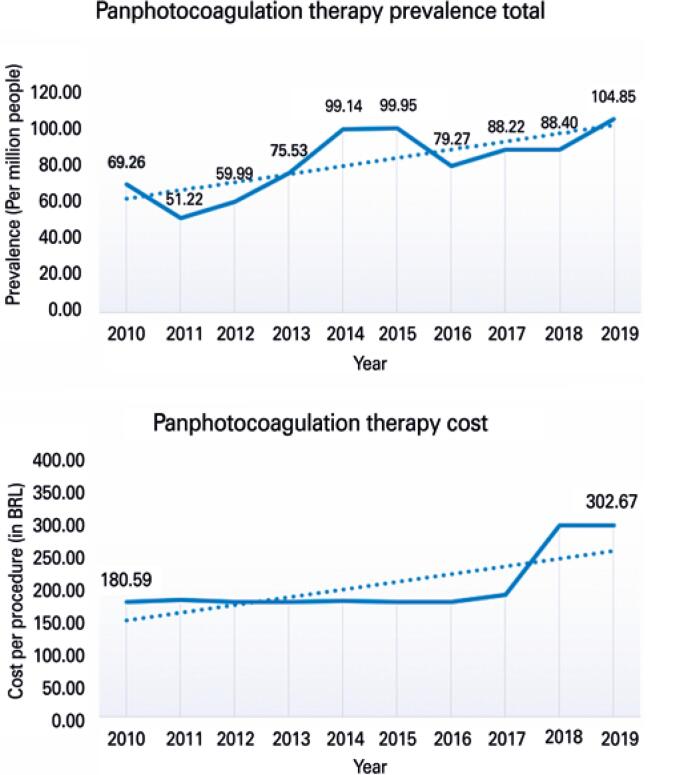
Panphotocoagulation procedure prevalence and cost trends from 2010 to 2019

Over the course of the 10-year period (2010 to 2019), 165,879 panphotocoagulation procedures were performed by the SUS. Figure 3 shows prevalence and cost trends per 1 million people during these years.

Trend analysis showed a significant increase in prevalence over time (p=0.005). Comparisons between 2019 and 2010 revealed a 51% difference. Significant changes (p=0.019) in procedure costs of service providers were observed only after 2018.

Table 3 shows the prevalence of panphotocoagulation procedures according to region.

**Table 3:** Prevalence (per million people) of panphotocoagulation procedures according to region

Year	Region					
	North	Northeast	Southeast	South	Midwest	All
2010	30.28	149.11	27.46	90.58	9.62	69.26
2011	52.82	40.53	45.07	104.61	21.35	51.22
2012	80.62	29.16	59.29	111.99	55.21	59.99
2013	106.88	56.08	61.58	129.04	87.96	75.53
2014	120.83	60.16	101.85	129.11	144.84	99.14
2015	111.24	64.63	108.10	134.55	104.52	99.95
2016	94.51	41.36	89.67	115.08	73.51	79.27
2017	109.20	33.86	98.49	132.83	118.58	88.22
2018	71.77	40.91	88.13	174.80	116.44	88.40
2019	90.72	53.37	110.21	191.25	113.15	104.85

An overall increase in prevalence was observed at different rates in different regions. Comparisons between 2010 and 2019 revealed an increase in prevalence in the Midwest, Southeast, North and South (1,077%, 301%, 200% and 111%, respectively), whereas a 64% decrease was observed in the Northeast region.

## DISCUSSION

Although widely used for investigations in other medical specialties,^(7–11)^ this is the first study to use the data extracted from DATASUS database for analysis in ophthalmology. Data derived from this system are essential for a broader understanding of the overall ocular health care scenario within the SUS, and to identify trends in therapeutic patterns in a given specialty, to inform healthcare managers and public health policy makers.

The study revealed an overall increase in the number of therapeutic procedures for retinal disorders over time, which may reflect improved access to healthcare within the national health system. Still, most procedures were performed in the Southeast region. The World Health Organization (WHO) recommends a minimum ratio of one ophthalmologist to 17 thousand inhabitants. According to the most recent census conducted by the Brazilian Council of Ophtalmology (*Conselho Brasileiro de Oftalmologia* - CBO), in 2019, the country has 20,455 practicing ophthalmologists, which results in ratio of one to 9,224 inhabitants – an improvement relative to the 2010 ratio of one to 17,620. However, data analysis per region revealed ratios of one to 7,599 in the Southeast, and one to 12,084 in the North.^(12)^ The disparities in the concentration of ophthalmologists in the country may impact the frequency of procedures per region.

As expected, intravitreal injections accounted for most of the increase in number of procedures. Intravitreal injection of VEGF inhibitors is the most prevalent ophthalmic procedure in developed countries and, according to recent reports, there has been an increase by more than 500-fold, between 2000 and 2012 (from 4,500 to 2.3 million injections per year).^(13,14)^ With population aging, an ever-increasing number of patients requiring intravitreal injections is to be expected worldwide.^(14–16)^

The relative frequency of laser procedures decreased in the Northeast region, an opposite trend relative to remaining regions. Laser therapies are often indicated for diabetic retinopathy, a condition strongly associated with disease duration.^(17)^ Hence, the longer the survival of diabetic patients, the higher the frequency of diabetic retinopathy. Interestingly, the mortality of diabetic patients in the Northeast is considerably higher than in the rest of the country. Therefore, the lower number of laser procedures in these patients may be explained by the lower frequency of cases of diabetic retinopathy, and/or by underdiagnosis, given diabetic individuals might develop other secondary complications, such as kidney failure and limb amputations, that lead to postponement of ocular care.^(18)^

Although the number of procedures increased over the course of the 10-year period, the amount paid to hospitals for delivering services was not readjusted. The price charged for intravitreal injections has not been significantly adjusted since 2010, and laser procedures have been minimally readjusted only after 2017. The system relies on a price list determined by the Ministry of Health for reimbursement of procedures to partner hospitals, regardless of service costs. The critical issue is the lack of flexibility and the absence of periodic price list readjustments, which translates into a chaotic financial scenario for partner hospitals.^(19–22)^ To ensure efficient cost management, the exact cost of procedures must be known and occasional variations must be accepted. Also, appropriate readjustments, at least according to inflation rates, must be made.

Data derived from DATASUS provide an overview of the system. However, some limitations must be emphasized. The program informs the number of procedures, but detailed information is lacking. For example, users cannot filter procedures according to medical indication. Therefore, it is not possible to associate a given procedure with a specific diagnosis. Also, in cases of intravitreal injections, there is no filter option per type of medication (*i.e*., aflibercept, ranibizumab or bevacizumab).

## CONCLUSION

There was an increasing trend towards performance of therapeutic procedures for retinal disorders covered by the Brazilian Public Health System, from 2010 to 2019, with expressive changes in the frequency of intravitreal injections, photocoagulation and panphotocoagulation procedures. Trends differ according to region of the country. Costs associated with aforementioned procedures did not change significantly over time.
