# Supplementary material for: Trends in treatment of retinal disorders in the Brazilian Public Health System over a 10-year period
Source: Einstein (Sao Paulo). 2021 Nov 24;19:eGS6616. doi: 10.31744/einstein_journal/2021GS6616 (PMC8664286; doi:10.31744/einstein_journal/2021GS6616)
Supplement: Translation

# Translation: Tendências no tratamento de distúrbios de retina no Sistema Único de Saúde brasileiro em um período de 10 anos*

**Authors:** Aline Nunes Ferraz, Rafael da Silva Lemos, Fernando Korn Malerbi, Rodrigo Brant, Arthur Gustavo Fernandes

**Affiliations:** 1 Universidade Federal de São São Paulo SP Brasil Escola Paulista de Medicina, Universidade Federal de São Paulo, São Paulo, SP, Brasil.

**Keywords:** Doenças retinianas/epidemiologia, Injeções intravítreas, Fotocoagulação, Vigilância em saúde pública, Epidemiologia, Custos de cuidados de saúde

## Abstract

**Objetivo:**

Investigar as tendências dos números e dos custos dos procedimentos de injeção intravítrea, fotocoagulação e panfotocoagulação realizados pelo Sistema Único de Saúde brasileiro, no período de 2010 a 2019.

**Métodos:**

Foram extraídos dados do Departamento de Informática do Sistema Único de Saúde acerca dos atendimentos associados aos procedimentos de injeção intravítrea, fotocoagulação e panfotocoagulação, realizados de 2010 a 2019. A prevalência de procedimentos e os custos foram analisados por ano e por região de atendimento. As tendências ao longo dos anos foram avaliadas por meio de modelos lineares generalizados. Valores de p=0,05 foram considerados estatisticamente significantes.

**Resultados:**

Em relação aos procedimentos específicos, houve aumento nas frequências de injeção intravítrea (1.088%), panfotocoagulação (51%) e fotocoagulação (37%), no comparativo de 2010 a 2019. A injeção intravítrea foi o procedimento que apresentou maior crescimento ao longo dos anos, porém não houve reajuste de custo significativo durante o período estudado.

**Conclusão:**

Ao longo de 10 anos, houve aumento significativo do número de atendimentos associados aos tratamentos de distúrbios da retina. Os custos relacionados aos procedimentos mostraram pouco reajuste ao longo dos anos. Embora o Departamento de Informática do Sistema Único de Saúde apresente algumas limitações, como imprecisões e falta de detalhamento em alguns procedimentos, esta é a ferramenta de dados disponível no Brasil para o acesso a pesquisa relacionada ao Sistema Único de Saúde e pode contribuir com informações da saúde ocular e os custos dos procedimentos.

## INTRODUÇÃO

Dentre os procedimentos indicados com maior frequência para o tratamento de patologias retinianas, destacam-se as injeções intravítreas, indicadas para casos de degeneração macular relacionada à idade (DMRI) e edema macular; a fotocoagulação, indicada para rupturas de retina, e a panfotocoagulação, usada em casos de retinopatia diabética proliferativa, edema macular e oclusão venosa da retina, entre outras enfermidades.^(1,2)^

A fotocoagulação retiniana terapêutica vem sendo praticada há mais de 50 anos.^(3)^ Esse tratamento é prescrito para todos os casos de microaneurisma e outros extravasamentos focais na região macular.^(4)^ Metanálise recente revelou que a fotocoagulação como terapia isolada pode reduzir as chances de perda de visão dentro de 1 a 3 anos quando comparada à não intervenção.^(3)^ A panfotocoagulação é definida como a ablação total da retina por *laser* combinada com pequenas ablações focais dispersas, que têm por objetivo prevenir a neovascularização adicional e o extravasamento em resposta à secreção de fator de crescimento endotelial vascular (VEGF) pela retina isquêmica.^(2)^

Embora os *lasers* tenham sido empregados por muitos anos como a principal modalidade terapêutica em pacientes com edema macular, o advento das injeções intravítreas revolucionou o tratamento dessa condição nas últimas décadas. Revisões recentes evidenciaram o sucesso do tratamento à base de injeções intravítreas na melhora da função visual em pacientes com edema macular. Nesses estudos, a maior frequência de injeção e a faixa etária mais baixa foram as duas principais variáveis relacionadas ao melhor prognóstico e a melhores desfechos terapêuticos.^(5)^

O Brasil é um dos poucos países do mundo a oferecer cobertura universal e gratuita de saúde financiada pelo governo federal, por meio do Sistema Único de Saúde (SUS), o que proporciona atendimento médico para toda a população em todas as especialidades médicas, desde os cuidados primários até os procedimentos mais complexos, disponibilizados em hospitais terciários, incluindo atenção oftalmológica. Todos os dados nacionais relacionados ao SUS são compilados no Departamento de Informática do Sistema Único de Saúde (DATASUS).^(6)^ Originalmente criado com fins administrativos, o DATASUS contém dados de todas as internações e procedimentos cobertos pelo SUS no Brasil. A versão disponível *on-line* protege as informações pessoais, porém dá acesso a dados referentes a códigos de procedimentos específicos, dados demográficos gerais, local e data de internação e custo dos procedimentos.^(7)^

## OBJETIVO

Investigar as tendências, em termos de número e custos, dos procedimentos de injeção intravítrea, fotocoagulação e panfotocoagulação realizados por meio do Sistema Único de Saúde brasileiro, de 2010 a 2019.

## MÉTODOS

O DATASUS foi utilizado como fonte primária de dados neste estudo. Essa base de dados é uma iniciativa do Governo Federal Brasileiro para coletar dados do sistema nacional de saúde e inclui dados de todos os hospitais públicos do país.

Todos os procedimentos de injeção intravítrea, fotocoagulação e panfotocoagulação realizados pelo SUS, de 2010 e 2019, foram incluídos neste estudo. A prevalência e o custo dos procedimentos foram analisados por ano e região.

O cálculo da prevalência incluiu o número total de procedimentos realizados no país como um todo em um determinado ano, com base no respectivo censo populacional, publicado pelo Instituto Brasileiro de Geografia e Estatística (IBGE).

As análises estatísticas foram realizadas utilizando o software Stata/SE *Statistical Software*, versão 14.0, 2015 (Stata Corp, College Station, Texas, Estados Unidos). Tabelas de frequência foram empregadas para análisedescritiva. As tendências de variações anuais foram avaliadas por meio de modelos lineares generalizados. Valores de p≤0,05 foram considerados estatisticamente significantes.

## RESULTADOS

Durante o período de 10 anos, de 2010 a 2019, 322.046 injeções intravítreas foram realizadas por meio do SUS. A figura 1 mostra as tendências de prevalência por 1 milhão de habitantes e o custo por procedimento ao longo dos anos.

**Figura 1:** Tendências de prevalência e custos dos procedimentos de injeção intravítrea de 2010 a 2019

A análise de tendências mostrou um aumento significativo (p<0,001) da prevalência ao longo dos anos. Comparando-se os anos de 2019 e 2010, a diferença foi de 1.088,0%. Não houve alteração significativa (p=0,233) de custo dos procedimentos nos serviços de saúde onde eles foram realizados durante o período de 10 anos.

A tabela 1 mostra a prevalência de acordo com a região onde o procedimento foi realizado.

**Tabela 1:** Prevalência (por milhão de habitantes) de injeções intravítreas por região

Ano	Região					
	Norte	Nordeste	Sudeste	Sul	Centro-Oeste	Todas
2010	4,99	22,56	29,30	41,94	32,89	27,47
2011	8,37	44,18	64,90	50,56	53,27	51,49
2012	9,20	66,56	97,84	56,68	82,54	74,68
2013	14,55	73,91	106,18	93,97	61,55	84,41
2014	16,44	124,02	160,43	124,22	122,89	130,12
2015	30,47	128,36	215,79	129,91	177,88	160,76
2016	40,87	131,18	282,36	173,28	189,93	197,53
2017	39,43	174,02	346,38	206,73	199,11	241,48
2018	46,91	184,60	382,19	275,53	235,55	272,63
2019	72,92	219,32	463,11	356,05	193,47	326,50

Observou-se aumento geral da prevalência em todas as regiões do país, embora em escalas distintas. Comparando-se os anos de 2010 e 2019, o aumento da prevalência nas regiões Sudeste, Norte, Nordeste, Sul e Centro-Oeste foi de 1.481%, 1.363%, 872%, 749% e 488%, respectivamente.

Durante o período de 10 anos, de 2010 a 2019, 1.113.516 fotocoagulações foram realizadas por meio do SUS. A figura 2 mostra as tendências de prevalência por 1 milhão de habitantes e o custo por procedimento ao longo dos anos.

**Figura 2:** Tendências de prevalência e custos dos procedimentos de foto-coagulação de 2010 a 2019

A análise de tendências mostra um aumento significativo (p<0,001) da prevalência ao longo dos anos. Comparando-se os anos de 2019 e 2010, a diferença calculada foi de 37%. Observaram-se alterações significativas (p=0,015) de custo dos procedimentos nas unidades de saúde onde eles foram realizados apenas a partir de 2018.

A tabela 2 mostra a prevalência dos procedimentos de fotocoagulação de acordo com a região onde o procedimento foi realizado.

**Tabela 2:** Prevalência (por milhão de habitantes) de procedimentos de fotocoagulação por região

Ano	Região					
	Norte	Nordeste	Sudeste	Sul	Centro-Oeste	Todas
2010	235,99	397,41	669,70	386,05	274,80	488,08
2011	177,32	373,68	756,73	474,47	409,97	535,75
2012	166,56	323,24	738,53	434,55	249,64	495,43
2013	133,81	281,44	768,05	471,83	224,46	496,84
2014	137,85	222,88	836,75	633,48	177,04	529,39
2015	177,85	193,27	918,93	618,74	208,64	559,45
2016	284,04	211,33	889,40	536,65	183,41	547,39
2017	346,46	222,46	891,41	618,83	182,72	568,28
2018	643,43	237,58	789,48	888,25	240,27	598,22
2019	715,53	257,59	907,37	931,51	262,13	667,48

Observou-se um aumento geral da prevalência em algumas regiões do país, embora em escalas distintas. Comparando-se os anos de 2010 e 2019, houve aumento da prevalência nas regiões Norte, Sul e Sudeste (203%, 141% e 35%, respectivamente) e queda nas regiões Nordeste e Centro-Oeste (35% e 5%, respectivamente).

Durante o período de 10 anos, de 2010 a 2019, 165.879 panfotocoagulações foram realizadas por meio do SUS. A figura 3 mostra as tendências de prevalência por 1 milhão de habitantes e o custo por procedimento ao longo dos anos.

**Figura 3:** Tendências de prevalência e custos dos procedimentos de panfotocoagulação de 2010 a 2019

A análise de tendências mostra aumento significante (p=0,005) da prevalência ao longo dos anos. Comparando-se os anos de 2019 e 2010, a diferença foi de 51%. Observaram-se alterações significativas (p=0,019) de custos dos procedimentos nos serviços de saúde onde eles foram realizados apenas a partir de 2018.

A tabela 3 mostra a prevalência de procedimentos de panfotocoagulação de acordo com a região onde o procedimento foi realizado.

**Tabela 3:** Prevalência (por milhão de habitantes) de procedimentos de panfotocoagulação por região

Ano	Região					
	Norte	Nordeste	Sudeste	Sul	Centro-Oeste	Todas
2010	30,28	149,11	27,46	90,58	9,62	69,26
2011	52,82	40,53	45,07	104,61	21,35	51,22
2012	80,62	29,16	59,29	111,99	55,21	59,99
2013	106,88	56,08	61,58	129,04	87,96	75,53
2014	120,83	60,16	101,85	129,11	144,84	99,14
2015	111,24	64,63	108,10	134,55	104,52	99,95
2016	94,51	41,36	89,67	115,08	73,51	79,27
2017	109,20	33,86	98,49	132,83	118,58	88,22
2018	71,77	40,91	88,13	174,80	116,44	88,40
2019	90,72	53,37	110,21	191,25	113,15	104,85

Observou-se aumento geral em diferentes regiões, em escalas distintas. Comparando-se os anos de 2010 e 2019, houve aumento da prevalência nas regiões Centro-Oeste, Sudeste, Norte e Sul (1.077%, 301%, 200% e 111%, respectivamente). Na região Nordeste, observou-se queda de 64%.

## DISCUSSÃO

Apesar do amplo uso para pesquisas em outras especialidades médicas,^(7–11)^ este foi o primeiro estudo a utilizar o DATASUS para análise na área de oftalmologia. Os dados derivados desse sistema são fundamentais para compreender melhor o panorama geral dos cuidados de saúde ocular disponibilizados pelo SUS e identificar tendências de padrões terapêuticos dentro de uma determinada especialidade, fornecendo informações para os gestores de saúde e os formuladores de políticas de saúde pública.

Este estudo revelou aumento geral do número de procedimentos terapêuticos para doenças retinianas ao longo dos anos, o que pode refletir um maior acesso a cuidados de saúde no sistema nacional. Ainda assim, a maioria dos procedimentos concentram-se na região Sudeste. A Organização Mundial da Saúde (OMS) recomenda uma proporção mínima de um oftalmologista por 17 mil habitantes e, de acordo com o censo mais recente realizado pelo Conselho Brasileiro de Oftalmologia (CBO), em 2019, o país contava com 20.455 oftalmologistas, o que corresponde a uma proporção de um para cada 9.224 habitantes, ou seja, resultado superior ao de 2010 (um para cada 17.620). Entretanto, na análise por região, essa proporção foi de um para cada 7.599 na região Sudeste e um para cada 12.084 naregião Norte.^(12)^ A disparidade de concentração de oftalmologistas no país pode influenciar na frequência de realização de procedimentos em diferentes regiões.

Conforme esperado, as injeções intravítreas foram as responsáveis pelo aumento mais evidente, em termos de número de procedimentos. Injeções intravítreas de inibidores de VEGF são os procedimentos oftalmológicos mais prevalentes em países desenvolvidos e, de acordo com relatos recentes, houve aumento de mais de 500 vezes entre 2000 e 2012 (de 4.500 para 2,3 milhões de injeções por ano).^(13,14)^ Dado o envelhecimento da população, espera-se aumento global crescente do número de pacientes que necessitem de injeções intravítreas.^(14–16)^

Observou-se queda da frequência relativa do número de procedimentos à base de *laser* na região Nordeste, que foi uma tendência oposta à das demais regiões. As terapias à base de *laser* são indicadas para tratamento da retinopatia diabética, uma alteração fortemente associada ao tempo de evolução da doença.^(17)^ Nesse sentido, quanto maior a sobrevida do paciente diabético, maior a frequência de retinopatia diabética. Curiosamente, a mortalidade dos pacientes diabéticos é muito mais alta no Nordeste do que nas demais regiões do país. Portanto, o número inferior de procedimentos à base de *laser* nesses pacientes pode ser explicado pelo menor número de casos de retinopatia diabética e/ou pelo seu subdiagnóstico, uma vez que os pacientes diabéticos podem ter outras complicações secundárias, como insuficiência renal ou amputações de membros, que possivelmente levam ao adiamento do cuidado ocular.^(18)^

Embora o número de procedimentos tenha aumentado ao longo do período de 10 anos, não houve reajuste do valor pago aos hospitais responsáveis pelo fornecimento do serviço. O valor cobrado pelas injeções intravítreas segue sem reajuste significativo desde 2010, e os procedimentos à base de *laser* só tiveram reajuste discreto a partir de 2017. O sistema adota um valor tabelado determinado pelo Ministério da Saúde para ressarcimento dos hospitais parceiros, independentemente do custo do serviço. Os principais pontos críticos são a falta de flexibilidade e a ausência de reajustes periódicos da tabela em questão, o que leva a uma situação financeira caótica nos hospitais envolvidos.^(19–22)^ A fim de garantir um bom gerenciamento de custos, é fundamental conhecer o valor gasto por procedimento, aceitar eventuais variações e realizar reajustes adequados, que acompanhem, ao menos, a inflação no país.

As informações obtidas a partir do DATASUS fornecem um panorama geral do sistema, entretanto, algumas limitações devem ser destacadas. O programa informa o número de procedimentos, mas não outros detalhes. Por exemplo, não é possível filtrar os procedimentos por indicação médica, o que impede a associação entre um determinado procedimento e um diagnóstico específico. Além disso, no caso das injeções intravítreas, não é possível filtrar por tipo de medicação (aflibercepte, ranibizumabe ou bevacizumabe).

## CONCLUSÃO

Notou-se aumento da tendência à realização de procedimentos voltados para o tratamento das doenças retinianas pelo Sistema Único de Saúde brasileiro, de 2010 a 2019, com alteração expressiva da frequência de realização de procedimentos de injeção intravítrea, fotocoagulação e panfotocoagulação. As tendências diferem de acordo com a região do país. Os custos associados aos procedimentos variaram pouco ao longo dos anos.
